# Mechanical origin of aftershocks

**DOI:** 10.1038/srep15560

**Published:** 2015-10-26

**Authors:** E. Lippiello, F. Giacco, W. Marzocchi, C. Godano, L. de Arcangelis

**Affiliations:** 1 Department of Mathematics and Physics and CNISM, Second University of Naples, 81100 Caserta, Italy; 2 Istituto Nazionale Geofisica Vulcanologia, 00143 Roma, Italy; 3 Department of Industrial and Information Engineering and CNISM Second University of Naples, 81031 Aversa (CE), Italy

## Abstract

Aftershocks are the most striking evidence of earthquake interactions and the physical mechanisms at the origin of their occurrence are still intensively debated. Novel insights stem from recent results on the influence of the faulting style on the aftershock organisation in magnitude and time. Our study shows that the size of the aftershock zone depends on the fault geometry. We find that positive correlations among parameters controlling aftershock occurrence in time, energy and space are a stable feature of seismicity independently of magnitude range and geographic areas. We explain the ensemble of experimental findings by means of a description of the Earth Crust as an heterogeneous elastic medium coupled with a Maxwell viscoelastic asthenosphere. Our results show that heterogeneous stress distribution in an elastic layer combined with a coupling to a viscous flow are sufficient ingredients to describe the physics of aftershock triggering.

The first empirical law for aftershock organisation in time dates back to Omori^1^,^2^ and states that the number of aftershocks *n*(*t*) decays as a power law with the time *t* from the mainshock, 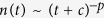. Many explanations for the Omori law have been proposed^3^ but a complete understanding of its origin is still lacking. The improvement in data acquisition and elaboration has contributed to identify^4^ the dependence of the characteristic time *c* in the Omori law on the rake angle λ. This angle indicates the direction of slip on the fault plane and can be related to the local level of differential stress *σ*_*D*_^5^. In particular under some assumptions, such as that faulting follows Mohr-Coulomb theory, *σ*_*D*_ is larger for 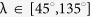 and smaller for 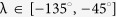. Within these hypotheses^4^,^5^,^6^, the rake angle can be, therefore, used to infer information on the differential stress acting on seismic faults, a quantity very difficult to measure directly. A similar dependence on λ has been previously observed^6^ for the parameter *b* in the Gutenberg-Richter (GR) law^7^, stating that the number of magnitude *m* earthquakes, 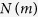, exponentially decreases with *m*, 
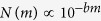
. These results offer new perspectives in earthquake-hazard analysis even if a precise physical interpretation of their origin is still lacking. Moreover, a clear identification of the mechanisms responsible for the *b* and *c* dependence on the differential stress might also contribute to a better understanding of the physics behind aftershock triggering, an issue still debated and controversial^8^,^9^,^10^. In this letter we show that also the size of the aftershock area depends on λ and we develop a coherent framework able to explain aftershock organisation in time, space and magnitude.

## Results

We first consider experimental data from the Southern California region and restrict the study to intermediate mainshock magnitudes 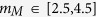. We identify aftershocks as events occurring within 10 min and in a circle of radius 3.3 km centered in the mainshock epicenter (see Methods).

We then define *L*_*a*_ as the average main-aftershock epicentral distance normalized by the typical size of the aftershock area^11^ 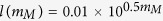 km. This choice, as shown in the Supplementary Information, ensures that the evaluation of *L*_*a*_ is not affected by variations of the *b* value. We evaluate *L*_*a*_ for all main-aftershock couples and finally stack sequences according to the rake angle λ of the mainshock in overlapping intervals of amplitude 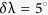. Only λ intervals containing at least 5 main-aftershock couples are included in the study. We have verified that results are not significantly affected by the interval value. We also evaluate the *c*-value for mainshock magnitudes 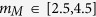. Details on the procedure to obtain *b* and *c* values are given in the Methods.

**Figure 1:**
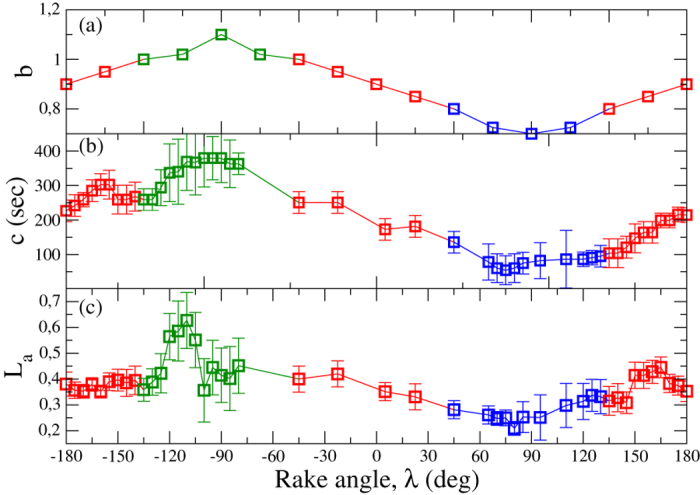
Statistical parameters of aftershock occurrence depend on the faulting style. (Upper panel) The *b* value in the GR law as function of the rake angle λ is extrapolated from the results of Schorlemmer *et al.*^6^ for Southern California. Different colors correspond to the Aki Richards convention for faulting styles: normal (green), strike-slip (red), thrust (blue) faults. (Central Panel) The *c* value of the Omori law as a function of the rake angle is evaluated for the Southern California region. Results refer to mainshock magnitudes in the range [2.5:4.5] and aftershocks with magnitudes larger than 2.4. Data are in very good agreement with results by Narteau *et al.*^4^ in their Fig. 1b. (Lower Panel) The normalized size of the aftershock area *L*_*a*_ as a function of the rake angle λ for the Southern California catalog. We have applied the same criterion as in the central panel to identify mainshocks and considered as aftershocks all events with 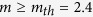 occurring within 3.3 km and 10 minutes after the mainshock occurrence.

**Figure 2:**
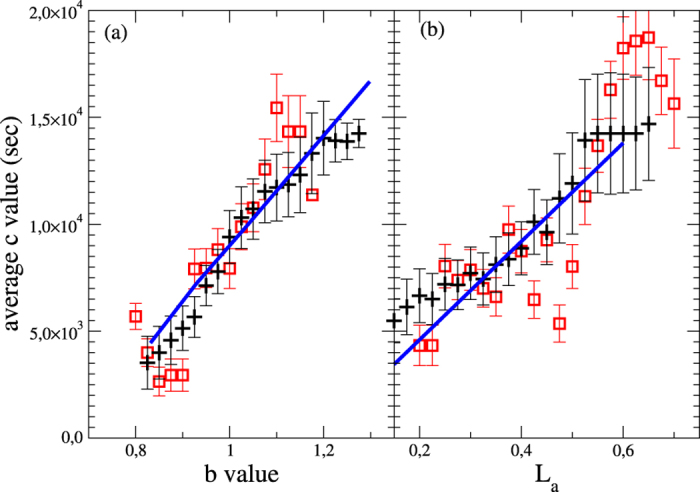
Positive correlations among statistical parameters of aftershock occurrence. (Left panel) Parametric plot of the *c* value as a function of *b*. Black pluses are obtained for mainshocks with magnitude in the range [2.5:4.5] for the Southern California by λ inversion of data in Fig. 1 (upper and central panel) vertically shifted by a factor 28. Open red squares are results for aftershocks triggered by 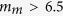 mainshocks averaged over other geographic areas. In this case only aftershocks with magnitude 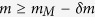, where *m*_*M*_ is the mainshock magnitude and *δm* = 4.5, are included in the analysis. The continuous blue line is the functional form of *c* vs *b* obtained from simulations of the numerical model (Fig. 4). The linear fit 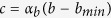 with 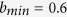 gives similar *α*_*b*_ for the tree data sets, 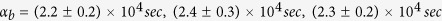, respectively. Results are obtained considering *b*-intervals of fixed amplitude 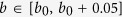 where *b*_0_ ranges from 0.5 to 1.3 in steps of 0.01. For each value of *b*_0_ the average value of all *c* values corresponding to the given interval is then considered. (Right panel) The same as in the left panel for the parametric plot of *c* vs *L*_*a*_. For each main-aftershock distance *δr* we evaluate 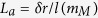 and group *L*_*a*_ in intervals 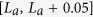 where *L*_*a*_ ranges from 0.1 to 0.65 in steps of 0.01. We plot the average *c* inside each *L*_*a*_ interval. The *α*_*L*_ obtained as best fit of the relation 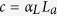 is very similar for the tree data sets, 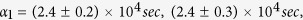 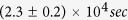, respectively.

Results for the average value of *c* and *L*_*a*_ as function of λ are plotted in Fig. 1. We also plot the dependence of *b* on λ, following the same procedure of ref. 6 without discrimination between aftershocks and mainshocks. Parametric plots of *c* vs *b* and *c* vs *L*_*a*_ indicate (Fig. 2) a proportionality among these quantities. This behaviour is also recovered for larger mainshocks and other geographic areas 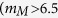 mainshocks from Southern California, Northern California, Japan, Alaska and 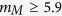 earthquakes in Italy). Details on the considered data sets can be found in the Supplementary Information. For each sequence we separately evaluate *b*, *c* and *L*_*a*_ and average over all sequences. The parametric plots (Fig. 2) indicate good agreement with data for Southern California 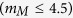, supporting positive correlations among *b*, *c* and *L*_*a*_ as a stable feature of seismicity. We wish to stress that the observed behaviour is not a spurious effect related to aftershock incompleteness^12^,^13^ (see Supplementary Information).

The dependence of *L*_*a*_ on λ leads to a better understanding of previous results on the *c* and *b* values^4^,^6^. Indeed, assuming that the fault area is proportional to the aftershock area, the seismic moment is proportional to 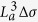, where Δ*σ* is the stress drop due to the mainshock. Therefore, for a given mainshock magnitude *m*_*M*_ (or seismic moment) a smaller value of *L*_*a*_ implies a larger Δ*σ*. Conversely, in regions where Δ*σ* is smaller, the same *m*_*M*_ can be only recovered if the stored elastic energy is distributed over a wider area. In the latter case, it is more probable to find several unstable regions scattered in space. This leads to a larger fraction of small aftershocks (a larger *b* value) and a longer temporal delay for the relaxation of all instabilities (a larger *c* value). The above description, therefore, predicts positive correlations among *b*, *c* and the spatial extent *L*_*a*_ of the aftershock area, experimentally observed. Since, under similar fault conditions, it is also reasonable to expect larger Δ*σ* in regions with larger *σ*_*D*_, the above argument also provides an explanation for the dependence of measured quantities on λ.

**Figure 3:**
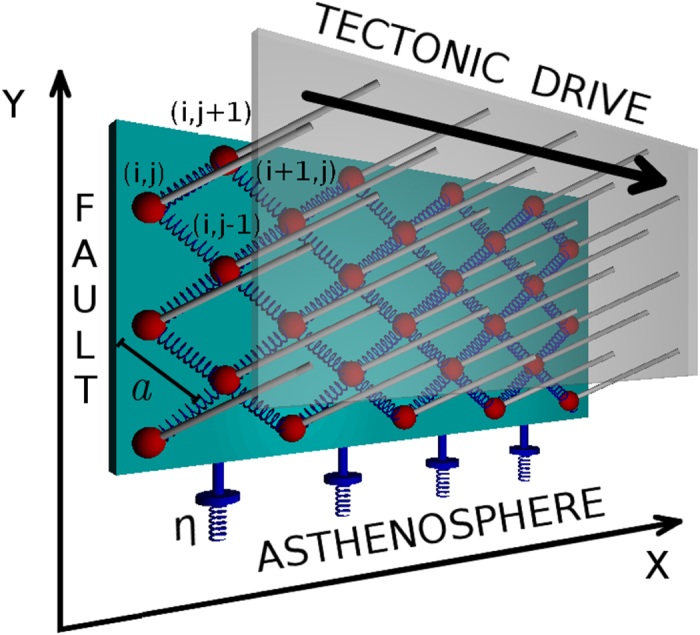
Schematic representation of the model for a seismic fault. The fault plane is an elastic layer of blocks connected by springs under a constant drive in the *x* direction. The plane is visco-elastically coupled to the Asthenosphere underneath according to the Maxwell rheology model. (Drawn by F.G.).

In the following we will show that the experimental statistical features of aftershocks are recovered in a model for a single seismic fault. The model can be extended to describe more realistic fault networks including secondary faults and different orientations with respect to the mainshock fault. Off-fault aftershocks can be expected in this case but their number would not be so relevant to affect the observed statistical results. The model implements three main ingredients. Ingredient 1 is the assumption that the fault plane is an elastic medium modelled as blocks interconnected by springs and subject to a stress with constant rate 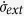 caused by the tectonic drive. More precisely we consider a tilted square lattice of spacing *a*. As soon as the local stress *σ*_*ij*_ exceeds the local static friction 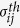, the block slips and stress is distributed to nearest neighbor blocks with the dynamic friction coefficient *μ*_*D*_ set to zero. An earthquake is represented by the ensemble of subsequent slips and its magnitude can be obtained from the size *S* of the slipping region. Ingredient 2 is the introduction of a heterogeneous local friction^14^,^15^,^16^ assuming that 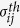 follows a quenched Gaussian distribution with mean 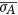 and standard deviation δσth = 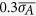. Randomness is also present in stress drops 
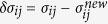
, where 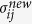 is randomly drawn, after each slip, from a Gaussian distribution of zero mean and standard deviation δσp = 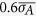. The precise value of 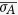 does not affect our results and the only free parameters are the standard deviations in the stress drops and in the friction levels. The hypothesis of randomness in stress relaxation reflects the existence of asperities in the fault plane leading to irregular local slips with the possibility that more blocks are simultaneously unstable. An earthquake starts at the most unstable site and involves neighboring blocks. Unstable blocks not involved in the event keep their local stress value that will be, eventually, relaxed at subsequent times. Ingredient 3 is the postseismic relaxation caused by the coupling between the elastic lithosphere of thickness *H*_*l*_ with a Newton viscous asthenosphere of thickness *H*_*a*_ (Fig. 3). We neglect vertical variations of the local strain and carry out a force balance for a given element in the lithosphere as in ref. 17. Approximating the viscous flow in the asthenosphere as a linear Couette flow we obtain the following equation for the local stress evolution


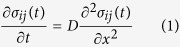


with the diffusion coefficient 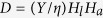
17,18 expressed in terms of the lithospheric Young modulus *Y* and the asthenospheric viscosity *η*. Model parameters and further details can be found in the Methods.

**Figure 4:**
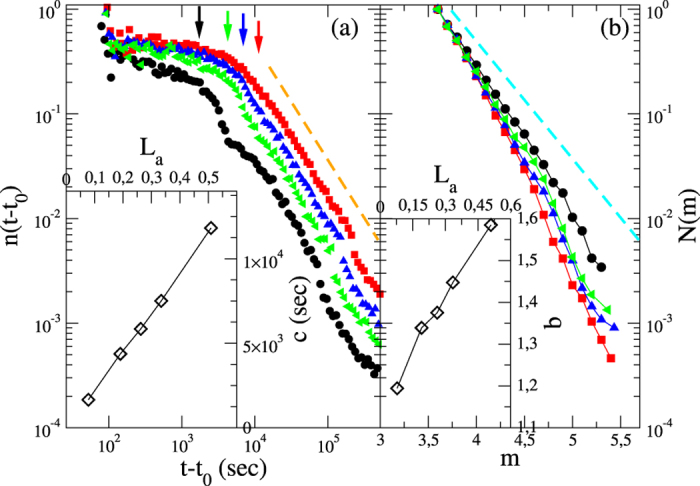
Statistics of aftershock sequences in the numerical model. (Left panel) The temporal decay of the number of aftershocks after a mainshock with magnitude 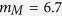 in the numerical model. Different colors correspond to different values of the initial shear stress 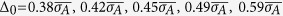 from right to left. Each curve is obtained by averaging over 10 different initial random configurations. Data exhibit different characteristic time scales *c* (indicated as coloured vertical arrows) for the onset of the power law decay for different Δ_0_. The orange dashed line indicates the Omori power law decay with an exponent *p* = 1.1. In the inset the parametric plot of *c* vs the size of the aftershock area *L*_*a*_. (Right panel) The magnitude distribution for aftershocks following a mainshock with magnitude 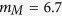. We adopt the same colour code of the left panel. The cyan dashed line indicates the exponential decay 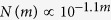 obtained in the whole numerical catalog. In the inset the parametric plot of *b* vs the size of the aftershock area *L*_*a*_.

**Figure 5:**
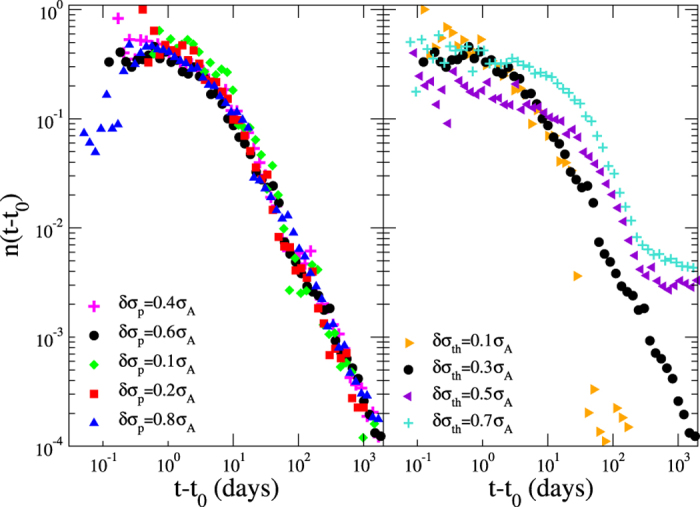
The role of model parameters. (Left panel) The distribution of the aftershock number as a function of the time from the mainshock for different values of *δσ*_*p*_. (Right panel) The distribution of the aftershock number as a function of the time from the mainshock for different values of *δσ*_*th*_.

In Supplementary Fig. 5 we plot the temporal evolution of a typical synthetic catalog. Aftershock sequences with patterns very similar to experimental data are clearly visible and the GR law, with a realistic value *b* = 1.1, is recovered under the assumption of local stress conservation. In order to identify the mechanisms for aftershock production we monitor the response of the system to a shear stress perturbation of the form 
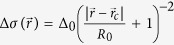
, where 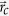 is the fault center, in absence of external stress rate 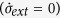. Starting from an initially stable configuration and applying this stress perturbation, the excess of stress is relaxed via a mainshock whose magnitude is tuned by Δ_0_ and *R*_0_. In the simplest version of the model (only ingredient 1) all the external stress is relaxed by the mainshock and aftershocks are not produced. The introduction of spatial heterogeneities (ingredient 2) leads to blocks that, not involved in the energy redistribution process during the main event, are still unstable after the mainshock occurrence. These blocks relax their energy at subsequent times and, as a consequence, aftershocks are triggered. Their activity is substantially constant in time and abruptly stops after a time delay *c* depending on the spatial extent of the perturbed region (*R*_0_). Viscoelastic relaxation (ingredient 3) leads to aftershock activity continuing after *c*. In this case the aftershock number decreases as a power law of time with an exponent close to *p* = 1.1 (Fig. 4a) in very good agreement with the Omori law of real seismic data. In Fig. 5 we investigate the influence on our results of the two free parameters: the standard deviations of the local friction distribution (*δσ*_*th*_) and the standard deviation in the value of the local stress after the stress-drop (*δσ*_*p*_). The variance *δσ*_*th*_ can be related to the number of asperities as well as to their size distribution within the fault. In our study we consider mainshocks with magnitude *m* = 6.3 and plot the number of events with *m* > 3.5 as a function of time from the mainshock for different choices of *δσ*_*p*_ (left panel) and *δσ*_*th*_ (right panel). We observe that results are substantially unaffected by *δσ*_*p*_ whereas different values of *δσ*_*th*_ lead to different results. More precisely, we observe that for 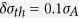, aftershocks follow the Omori decay up to a given time when their number abruptly decreases to zero and the power law regime is no longer observed. On the other hand, for large values of *δσ*_*th*_ a constant, roughly stationary, (*background*) seismic activity is superimposed to the aftershock decay rate. In this case the power law decay regime reduces to less than one decade before approaching the constant background rate with a quite stable exponent *p*. We have also explored the influence of different values of *μ*_*D*_ > 0 which only affects the level of the background rate, becoming larger for larger *μ*_*D*_. Conversely, the aftershocks decay is not affected by *μ*_*D*_ and the Omori parameters *p* and *c* are *μ*_*D*_ independent.

We wish to stress that many spring-block models, based on ingredients 1 and 2, have been proposed in the literature even implementing more complex, time dependent or state dependent, friction laws^19^,^20^,^21^,^22^,^23^. Even if these models exhibit non-trivial temporal patterns, they are not able to reproduce aftershock occurrence in agreement with experimental data. This observation, together with our findings (Figs 4 and 5), of numerical aftershocks following the Omori law, confirm previous results concerning the central role of viscous coupling (ingredient 3) for aftershock triggering^19^,^24^,^25^,^26^. Similar results can be also recovered by the Jagla model^27^,^28^,^29^ where *δσ*_*ij*_ is constant, the friction thresholds 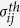 are randomly updated after each slip and a different equation for stress relaxation is implemented.

To explore the role of the level of differential stress in the aftershock organisation, we analyse different values of 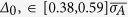, keeping the mainshock magnitude 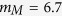 fixed. As a consequence, the value of *R*_0_ is changed accordingly, with larger Δ_0_ corresponding to smaller *R*_0_. In Fig. 4a we plot the number of events 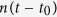 with 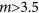 as function of time from the mainshock. Each curve is obtained by averaging over 10 different initial random configurations. The Omori law is observed for each value of Δ_0_ and the *c* value (indicated by vertical arrows) decreases for increasing stress levels (larger Δ_0_). We also find that the *b* value in the GR law is a decreasing function of Δ_0_ (Fig. 4b), leading to positive correlations between the parameters *c* and *b*. Fig. 4 also indicates that larger *c* values correspond to larger values of *R*_0_, predicting a positive correlation between *c* and the size of the aftershock area *L*_*a*_. The parametric plots (insets of Fig. 4) reproduce the same linear trends observed in experimental data and therefore, by assuming a given relationship between Δ_0_ and λ, the experimental results in Fig. 1 are reproduced by the numerical model. The agreement between experimental and numerical results indicates that the heterogeneous stress distribution in an elastic layer combined with a viscous coupling are necessary and sufficient ingredients to describe aftershock occurrence.

## Methods

### Mainshocks and aftershocks identification

We apply a space-time window criterion to discriminate between mainshocks and aftershocks^8^: An event is identified as a mainshock if a larger earthquake does not occur in the previous *y* days and within a distance *L*. In addition, a larger earthquake must not occur in the selected area in the following *y*_2_ days. We use typical values *L* = 100 km, *y* = 3 and *y*_2_ = 0.5. Aftershocks are all events with magnitude larger than *m*_*a*_ = 2.4 occurring in the subsequent time interval 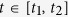 and within a circle of radius *R* from the mainshock epicenter. The sets of parameters *t*_1_, *t*_2_ and *R* are listed in Table 2 of the Supplementary Information.

For each sequence the *c* value is obtained by means of a maximum likelihood maximization routine keeping *p* = 1.1 fixed for all sequences. We finally average *c* over all sequences belonging to a given λ interval. In the case of large mainshocks 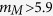, the *c* value is obtained keeping *p* = 1.1 fixed for all sequences and considering only aftershocks with magnitude 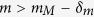, with 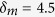. Only sequences with at least 200 aftershocks have been included in the study. Results for different choices of *δ*_*m*_ are discussed in the Supplementary Information. The *b* value is obtained by means of the maximum likelihood estimation^30^ 
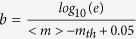
 with 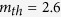. In all cases, other parameter values provide similar results.

### The spring-block model

We represent the fault plane as an elastic medium made by blocks on a tilted square lattice of spacing *a* interconnected by springs. Blocks are under the action of a uniform tectonic drive 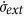 in the *x*-direction, and are coupled to a Maxwell viscoelastic layer (Fig. 3). In the hypothesis that the slip is much smaller than *a* and that the stress redistribution after the slip is instantaneous, the system evolution can be expressed only in terms of *σ*_*ij*_. The simulation proceeds as follows: We randomly assign a quenched threshold 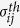 and initial condition 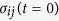, at each site. Local stresses are then updated according to Eq.(1) whose discretized form, including the tectonic drive, reads


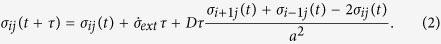


If at some time *t* one or more sites are unstable 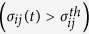, the stress at the site with the largest values of 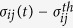 is updated to a random value, each time extracted from a Gaussian distribution. The relaxed stress 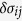 is uniformly distributed to the four 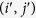 nearest neighbor blocks 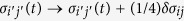, obeying local stress conservation. If at least one of these blocks is unstable, a further stress relaxation occurs and the process is iterated. The redistribution of stress stops as soon as no further nearest neighbor block is unstable. The whole process is considered instantaneous and afterwards the temporal evolution is iterated according to Eq.(2). The magnitude of an earthquake occurring at time *t* is evaluated from the number of blocks *N*_*b*_ that simultaneously slip via the empirical relation 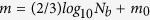. We fix 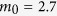 and according to the empirical relationship^31^ between the magnitude and the rupture area, this corresponds to fixing the lattice spacing to 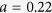 Km. Implementing typical values^19^ for 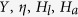 one has 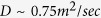 and therefore we fix the time step of numerical integration 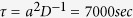. Stress is expressed in units of the average value 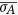 whose value is irrelevant.

## Additional Information

**How to cite this article**: Lippiello, E. *et al.* Mechanical origin of aftershocks. *Sci. Rep.* **5**, 15560; doi: 10.1038/srep15560 (2015).

## Supplementary Material

Supplementary Materials

## References

[b1] Omori F. On the aftershocks of earthquakes. J. College of Science, Imp. Univ. Tokyo 7, 111–200 (1894).

[b2] Utsu T. Aftershocks and earthquake statistics. J. Fac. Sci. Hokkaido Univ. Ser. VII 3, 379441 (1965).

[b3] Freed A. M. Earthquake triggering by static, dynamic, and postseismic stress transfer. Annu. Rev. Earth Planet. Sci. 33, 335–367 (2005).

[b4] Narteau C., Byrdina S., Shebalin P. & Schorlemmer D. Common dependence on stress for the two fundamental laws of statistical seismology. Nature 462, 642–645 (2009). 19956258 10.1038/nature08553

[b5] Sibson R. H. Frictional constraints on thrusts, wrench and normal faults. Nature 249, 542–544 (1974).

[b6] Schorlemmer D., Wiemer S. & Wyss M. Variations in earthquake-size distribution across different stress regimes. Nature 437, 539–542 (2005). 16177788 10.1038/nature04094

[b7] Gutenberg B. & Richter C. F. Frequency of earthquakes in california. Bull. Seismol. Soc. Am. 34, 185–188 (1944).

[b8] Felzer K. & Brodsky E. Decay of aftershock density with distance indicates triggering by dynamic stress. Nature 441, 735–738 (2006). 16760974 10.1038/nature04799

[b9] Lippiello E., de Arcangelis L. & Godano G. Role of static stress diffusion in the spatiotemporal organization of aftershocks. Phys. Rev. Lett. 103, 038501–4 (2009). 19659324 10.1103/PhysRevLett.103.038501

[b10] Richards-Dinger K., Stein R. S. & Toda S. Decay of aftershock density with distance does not indicate triggering by dynamic stress. Nature 467, 583–587 (2010). 20882015 10.1038/nature09402

[b11] Utsu T. & Seki A. A relation between the area of aftershock region and the energy of mainshock. J. Seismol. Soc. Jpn. 7, 223–240 (1954).

[b12] Helmstetter A., Kagan Y. & Jackson D. Comparison of short- term and time-independent earthquake forecast models for southern california. Bull. Seism. Soc. Am. 96 **(1)**, 90–106 (2006).

[b13] Lippiello E., Godano C. & de Arcangelis L. The earthquake magnitude is influenced by previous seismicity. Geophys. Res. Lett. 39, L053091–5 (2012).

[b14] Fisher D. S., Dahmen K., Ramanathan S. & Ben-Zion Y. Statistics of earthquakes in simple models of heterogeneous faults. Phys. Rev. Lett. 78, 4885–4888 (1997).

[b15] Zöller G., Hainzl S., Holschneider M. & Ben-Zion Y. Aftershocks resulting from creeping sections in a heterogeneous fault. Geophysical Research Letters 32, L033081–4 (2005).

[b16] Kazemian J., Dominguez R., Tiampo K. & Klein W. Spatial heterogeneity in earthquake fault-like systems. Pure and Applied Geophysics 1–11, 10.1007/s00024-014-0843-6, (2014).

[b17] Turcotte D. L. & Schubert G. Geodynamics (Cambridge University Press, Cambridge, 679–682 (2002).

[b18] Rydelek P. & Sacks I. Asthenospheric viscosity and stress diffusion: a mechanism to explain correlated earthquakes and surface deformations in ne japan. Geophys. J. Int. 100, 39–58 (1990).

[b19] Pelletier J. D. Spring-block models of seismicity: Review and analysis of a structurally heterogeneous model coupled to a viscous asthenosphere. Geocomplexity and the Physics of Earthquakes *edited by* Rundle J. B., Turcotte D. L. & Klein W. 25–41 (2000).

[b20] Lippiello E., de Arcangelis L. & Godano C. Memory in self-organized criticality. EPL (Europhysics Letters) 72, 678 (2005).

[b21] Ohmura A. & Kawamura H. Rate- and state-dependent friction law and statistical properties of earthquakes. EPL (Europhysics Letters) 77, 69001 (2007).

[b22] Clancy I. & Corcoran D. State-variable friction for the burridge-knopoff model. Phys. Rev. E 80, 016113 (2009). 10.1103/PhysRevE.80.016113 19658780

[b23] Kawamura H., Hatano T., Kato N., Biswas S. & Chakrabarti B. K. Statistical physics of fracture, friction, and earthquakes. Rev. Mod. Phys. 84, 839–884 (2012).

[b24] Hainzl S., Zöller G. & Kurths J. Similar power laws for foreshock and aftershock sequences in a spring-block model for earthquakes. J. Geophys. Res. : Solid Earth 104, 72437253 (1999).

[b25] Narteau C., Shebalin P., Hainzl S., Zöller G. & Holschneider M. Emergence of a band-limited power law in the aftershock decay rate of a slider-block model. Geophysical Research Letters 30, 22-1–22-4 (2003).

[b26] Jagla E. A., Landes F. M. C. P. & Rosso A. Viscoelastic effects in avalanche dynamics: A key to earthquake statistics. Phys. Rev. Lett. 112, 174301 (2014). 24836251 10.1103/PhysRevLett.112.174301

[b27] Jagla E. A. Realistic spatial and temporal earthquake distributions in a modified olami-feder-christensen model. Phys. Rev. E 81, 046117 (2000). 10.1103/PhysRevE.81.046117 20481796

[b28] Jagla E. A. & Kolton A. B. A mechanism for spatial and temporal earthquake clustering. J. Geophys. Res.: Solid Earth 115, B05312 (2010).

[b29] Aragon L. E., Jagla E. A. & Rosso A. Seismic cycles, size of the largest events, and the avalanche size distribution in a model of seismicity. Phys. Rev. E 85, 046112 (2012). 10.1103/PhysRevE.85.046112 22680543

[b30] Guo Z. & Ogata Y. Statistical relations between the parameters of aftershocks in time, space, and magnitude. J. Geophys. Res.: Solid Earth 102, 28572873 (1997).

[b31] Wells D. L. & Coppersmith K. J. New empirical relationships among magnitude, rupture length, rupture width, rupture area, and surface displacement. Bull. Seismol. Soc. Amer. 84, 974–1002 (1994).

