# Supplementary material for: Mechanical origin of aftershocks
Source: Sci Rep. 2015 Oct 26;5:15560. doi: 10.1038/srep15560 (PMC4620523; doi:10.1038/srep15560)
Supplement: Supplementary Materials [file srep15560-s1.pdf]

Mechanical origin of aftershocks: Supplementary Information

E. Lippiello
Department of Mathematics and Physics, Second University of Naples,
Via Vivaldi 43, 81100 Caserta, Italy & Kavli Institute for Theoretical Physics,
Kohn Hall, University of California, Santa Barbara, CA 93106-4030 USA

F. Giacco & C. Godano
Department of Mathematics and Physics, Second University of Naples, Via Vivaldi 43, 81100 Caserta, Italy

W. Marzocchi
INGV, Via di Vigna Murata 605, 00143 Roma, Italy.

L. de Arcangelis
Department of Industrial & Information Engineering and CNISM,
Second University of Naples, Aversa (CE), Italy & Kavli Institute for Theoretical Physics,
Kohn Hall, University of California, Santa Barbara, CA 93106-4030 USA

TABLE I. Details on the experimental catalogs.

Geographic area	Temporal interval T_{min} , T_{max}	Latitude interval Lat_{min} , Lat_{max}	Longitude Interval Lon_{min} , Lon_{max}	Number of $m_M > 6.5$ mainshocks
Southern California [1]	1/1/1984 , 12/31/2002	31 , 37.3	-121.7 , - 113.8	3
Northern California [2]	1/1/1984 , 12/31/2011	35.3, 42.4 34	-126 , 1116	3
Japan mainland [3]	1/1/1966 , 01/30/2011	30 , 45.5	128.3 , 147	6
Alaska [4]	1/1/1990 , 01/31/2013	50 , 71	130 , 188	1
Italy [5]	1/1/2002 , 12/31/2012	35 , 48	6 , 19	2

TABLE II. Parameters in the declustering procedure

Measured quantity	t_1	t_2	R
c for $m_M \in [2.5, 4.5]$	100 sec	1 day	0.02 km
L_a for $m_M \in [2.5, 4.5]$	0 sec	600 sec	$10^{0.5m_M-2}$ km
c, L_a for $m_M \geq 5.9$	30 min	100 days	$10^{0.5m_M-2}$ km

I. DETAILS ON USED DATA SETS.

The study on intermediate mainshock magnitude $m_M \in [2.5, 4.5]$ is performed on the relocated catalog for Southern California [1].

Details on the catalogs used for the study on other geographic areas for large mainshocks $m_M > 6.5$ can be found in Table 1. In Table 1 we also report the number of mainshocks identified for each data set. For the Italian region we select the two largest $m_M \geq 5.9$ mainshocks in the catalog. In all cases we restrict to events with depth smaller than 80 km.

In Table 2 we report the parameters t_1, t_2 and R implemented in the aftershock identification procedure. We have used three different parameter sets, one for aftershock identification after large mainshocks, one for the measure of c in mainshock sequences with $m_m \in [2.5, 4.5]$ and one for the measure of L_a in mainshock sequences with $m_m \in [2.5, 4.5]$. The different choice of parameter sets is motivated by the observation that aftershock rate is much larger than the background seismicity for several days after large shocks. In particular, for $m_m > 6.5$ the contamination by background events is very weak and one can consider also large temporal intervals extending up to $t_2 = 100$ days where the Omori law is clearly observed. Conversely, also in a temporal interval of few hours after small mainshocks $m_m \leq 4.5$, the contribution of background seismicity cannot be neglected. To fix this problem, since in the evaluation of c we are interested in the aftershock temporal organization, we used a sufficiently large temporal interval $t_2 = 1$ day but a narrow spatial range ($R = 0.02$ km). This is exactly the same spatio-temporal range considered in ref.[4]. On the other hand, in the evaluation of L_a we are interested in the aftershock distribution in space and in this case we have considered a larger R ($R = l(m_m)$) but a very small temporal interval, $t_2 = 10$ minutes. Within these spatio-temporal ranges, we are quite confident that the influence of background events is never relevant. The choice of t_1 is motivated by data incompleteness in the first part of main-aftershock sequences (see Sec.III). The observed incompleteness is present on larger time scales for larger mainshocks and this implies different values of t_1 for different data sets.

II. THE ROLE OF THE λ-DEPENDENCE OF THE b-VALUE ON THE λ-DEPENDENCE OF THE AFTERSHOCK AREA

This section is devoted to show that the observed dependence of L_a on λ is not an indirect consequence of the dependence of the b-value on λ. Let us introduce the main-aftershock epicentral distance δr and and let us indicate with $\langle \delta r(m, \lambda) \rangle$ the average main-aftershock epicentral distance for a mainshock with magnitude m and rake angle λ. Let us consider two mainshocks m_1, m_2 with same magnitude $m_1 = m_2 = m$ and different rake angles $\lambda_1 \neq \lambda_2$. Under the assumption that the aftershock spatial distribution is λ independent, the dependence of $\langle \delta r(m, \lambda) \rangle$ on the rake angle is only through the mainshock magnitude. Therefore, since $m_1 = m_2$, we would get $\langle \delta r(m_1, \lambda_1) \rangle = \langle \delta r(m_2, \lambda_2) \rangle = \Gamma(m)$, where $\Gamma(m) \sim l(m) \sim 10^{0.5m}$ is a monotonic increasing function of m [6]. By averaging now over mainshock magnitudes in the range $m \in [2.5, 4.5)$ we obtain

$$\overline{\langle \delta r(\lambda) \rangle} = \int_{2.5}^{4.5} dm \langle \delta r(m, \lambda) \rangle P(m, \lambda) \tag{1}$$

where the mainshock magnitude distribution $P(m)$ follows the Gutenberg-Richter law $P(m) \propto 10^{-b(\lambda)m}$. Under the above hypothesis of an aftershock spatial distribution independent of λ, we obtain

$$\overline{\langle \delta r(\lambda) \rangle} \propto \int_{2.5}^{4.5} dm \Gamma(m) 10^{-b(\lambda)m}. \tag{2}$$

Therefore the only dependence on λ is through $b(\lambda)$ and, in this case, $\overline{\langle \delta r(\lambda) \rangle}$ is a decreasing function of b. Comparing the above prediction with data from real seismic catalogs (red open squares in Suppl.Fig.1) we find the opposite dependence: $\overline{\langle \delta r(\lambda) \rangle}$ is an increasing function of b. Experimental data therefore indicate that also $\langle \delta r(m, \lambda) \rangle$ explicitly depends on λ and therefore also the aftershock spatial organization is affected by the rake angle.

In order to separate the dependence on λ of $\langle \delta r(m, \lambda) \rangle$ from the one of $b(\lambda)$, in the manuscript we have considered the quantity

$$L_a = \int_{2.5}^{4.5} dm \frac{\langle \delta r(m, \lambda) \rangle}{l(m)} P(m, \lambda). \tag{3}$$

Indeed, from the experimental scaling relation $\Gamma(m) \sim l(m)$, it is reasonable to expect that $\langle \delta r(m, \lambda) \rangle / l(m)$ weakly depends on m and, since $\int dm P(m, \lambda) = 1$, L_a is substantially $b(\lambda)$ independent.

The above arguments can be explicitly tested by means of numerical simulations. In particular we consider synthetic catalogs generated according to the Epidemic Type Aftershock Sequence (ETAS) model [7, 8]. This model describes seismic occurrence as the superposition of independent mainshocks (the ancestors) which trigger their offspring (the aftershocks). Furthermore, every aftershock can trigger its own offspring leading to an epidemic structure. Well established empirical laws are implemented for the main-aftershock spatio-temporal correlations. In particular here we use typical model parameters and spatial kernels as in ref. [9]. We assume that the only parameter depending on λ is b and all the other parameters are λ independent. We therefore generate different catalogs, each one corresponding to a different value of b. To each catalog we apply the main-aftershock selection procedure used for the experimental catalog. Results plotted in Suppl.Fig.1 (black circles in the upper panel) confirm our prediction that $\overline{< \delta r >}$ is a decreasing function of b, an opposite trend with respect to experimental results (red squares). In the lower panel of Suppl.Fig.1 we present the comparison between the dependence of L_a on b in the numerical catalog (black circles) and the experimental one (red squares). As expected the numerical L_a is substantially b independent indicating that the behavior of L_a in experimental data is the correct measure of the λ dependence of the aftershock area.

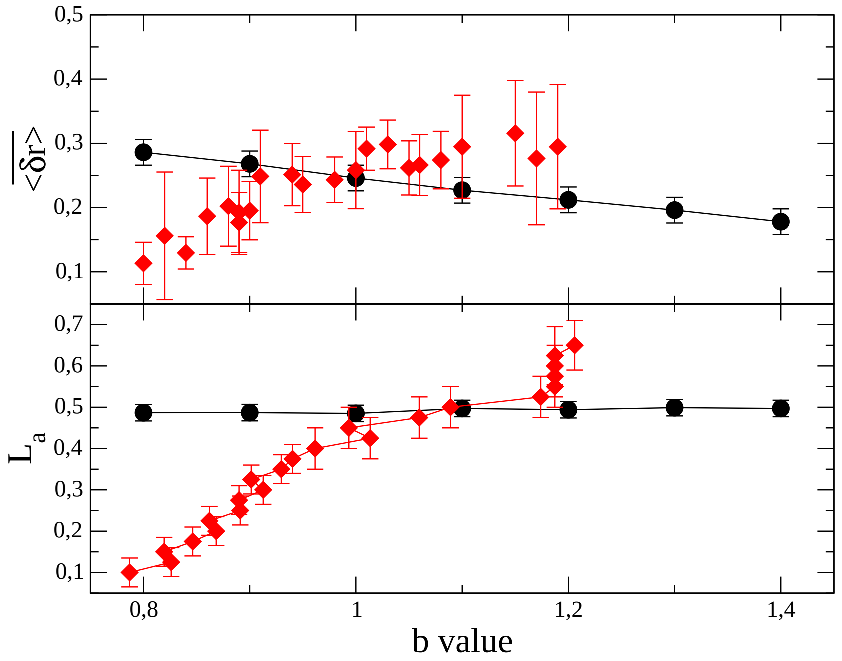

FIG. 1.
(Upper Panel) Parametric plot of $\overline{\langle \delta r(\lambda) \rangle}$ defined in Eq.(2) as function of the b-value for experimental results (red squares) and numerical data (black circles) from the ETAS model simulation. (Lower panel) The same as in the upper panel for the "aftershocks area" L_a defined in Eq.(3) and discussed in the manuscript.

III. THE EFFECT OF SHORT TERM AFTERSHOCK INCOMPLETENESS ON EXPERIMENTAL FINDINGS

This section is devoted to show that the observed correlations among b and c and L_a, observed in experimental data sets, cannot be spurious effects related to catalog incompleteness. Because of technical problems experimental catalogs are incomplete in particular in the early part of aftershock sequences following large shocks [10]. This short term aftershock incompleteness (STAI) leads to deviations from the GR law. More precisely, one can define a crossover magnitude $m_c(\Delta t)$, where Δt is the time from the mainshock so that $P(m)$ follows the GR relation for $m > m_c(\Delta t)$, whereas $P(m)$ exhibits a flat behavior for $m < m_c(\Delta t)$. The crossover magnitude $m_c(\Delta t)$ exhibits a regular trend consistent with

$$m_c(\Delta t) = m_0 - \delta \log(\Delta t) - m_1 \quad (4)$$

where m_0 is the mainshock magnitude, δ and m_1 are fitting parameters. Helmstetter et al. [11] find $\delta = 0.75$ and $m_1 = 4.5$. Some authors interpret the c value for the onset of the power law decay in the Omori law as an artifact related to STAI. More precisely, indicating with m_a the lower magnitude for aftershock detection, one can identify c with the time such that $m_c(c) = m_a$. According to this interpretation, the c obtained from experimental catalogs is a quantity caused by technical problems and does not contain any physical information. We wish to stress that, even if a deficit of small events at the beginning of a seismic sequence leads to longer c values, this cannot be responsible for the observed positive correlations between b and c. Indeed, let us indicate with b_0 and c_0 the "true" values of b and c, respectively, for a complete data set and with b_m and c_m their measured values affected by incompleteness. It is simple to show that STAI not only leads to $c_m > c_0$, as explained above, but also to an average value of m larger than the "true" one producing a measured $b_m \propto (\langle m \rangle - m_{th} - 0.05)^{-1}$ systematically smaller than b_0. As a consequence, for fixed b_0 and c_0, STAI produces c_m and b_m that are inversely correlated, as opposed to the trend observed in experimental data.

To confirm this interpretation we have generated numerically aftershock sequences by means of the ETAS model following the standard procedure outlined in Sec.II and assuming fixed values $b_0 = 1.1$, $p = 1.1$ and $c_0 = 2500$ sec. Each sequence contains on average 25000 aftershocks. To simulate the effect of incompleteness, we remove from the simulated catalogs aftershocks with magnitude $m < m_c(\Delta t)$ according to Eq.(4). We set $\delta = 0.75$ and consider different choices for m_1. Smaller values of m_1 lead to larger incompleteness and, as expected, produce larger c and smaller b values. The parametric plot c vs b obtained for different values of $m_1 \in [0.5, 2]$ is represented in Suppl. Fig.2. The functional dependence of c vs b is opposite to the one experimentally observed.

To further support that the correlation between c and b is a physical effect, not related to spurious incompleteness, we perform the same study as in Fig.2 of the manuscript for different values of the aftershock thresholds $m_a = m_M - \delta_M$. Incompleteness

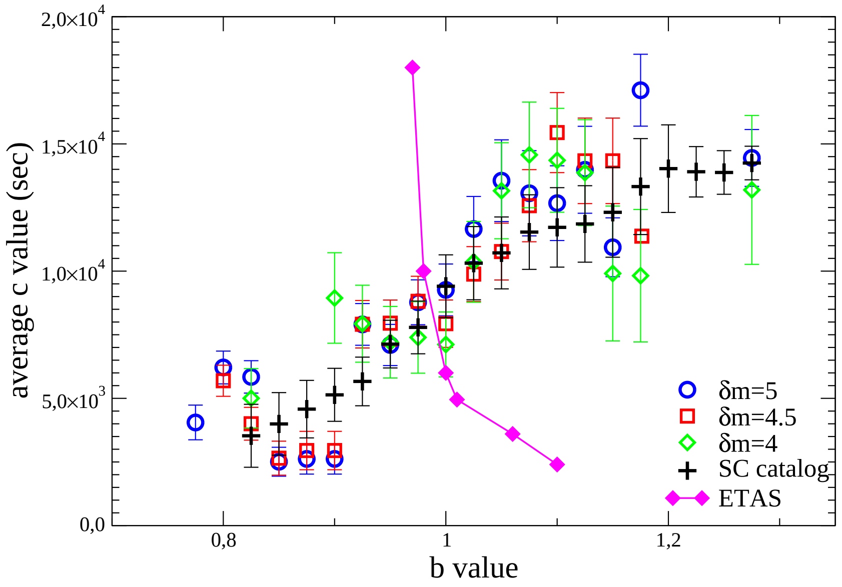

FIG. 2.
Parametric plot of the c value as function of b. Black pluses are for mainshocks with magnitude in the range $[2.5 : 4.5]$ in Southern California. Open symbols are for aftershocks with magnitude $m \geq m_M - \delta m$, triggered by large mainshocks averaged over other geographic areas, for different values of δm: $\delta m = 4$ (green triangles), $\delta m = 4.5$ (red squares) and $\delta m = 5$ (blue circles). Filled magenta diamonds are results of numerical simulations of the ETAS model with STAI artificially implemented.

is expected to be more pronounced for larger δ_M. Conversely, Suppl. Fig.2 indicates that the linear relationship between c and b weakly depends on δ_M and therefore it is weakly affected by STAI. The same analysis (Suppl. Fig.3) for the parametric plot c vs L_a for different choices of δ_M also supports the correlation between c and L_a as a physical feature of experimental aftershock sequences.

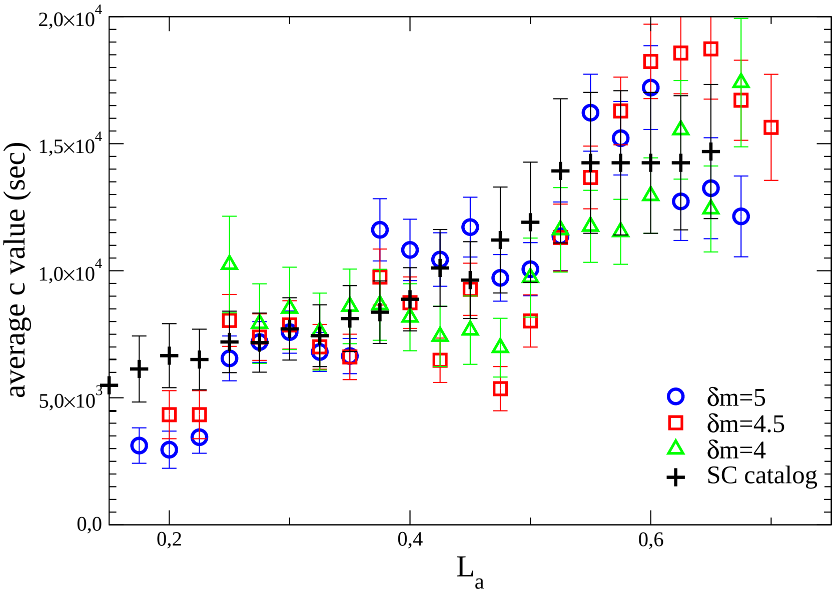

FIG. 3.
Parametric plot of the c value vs the size of the aftershock area L_a. Black pluses are for mainshocks with magnitude in the range $[2.5 : 4.5]$ in Southern California. Open symbols are results for aftershocks with magnitude $m \geq m_M - \delta m$, triggered by large mainshocks averaged over other geographic areas, for different values of δm: $\delta m = 4$ (green triangles), $\delta m = 4.5$ (red squares) and $\delta m = 5$ (blue circles).

IV. MAGNITUDE-FREQUENCY DISTRIBUTION AND AFTERSHOCKS IN THE NUMERICAL MODEL UNDER TECTONIC DRIVE

In supplem. Fig.4 we plot results of numerical simulations of the model described in Fig.3 of the manuscript.

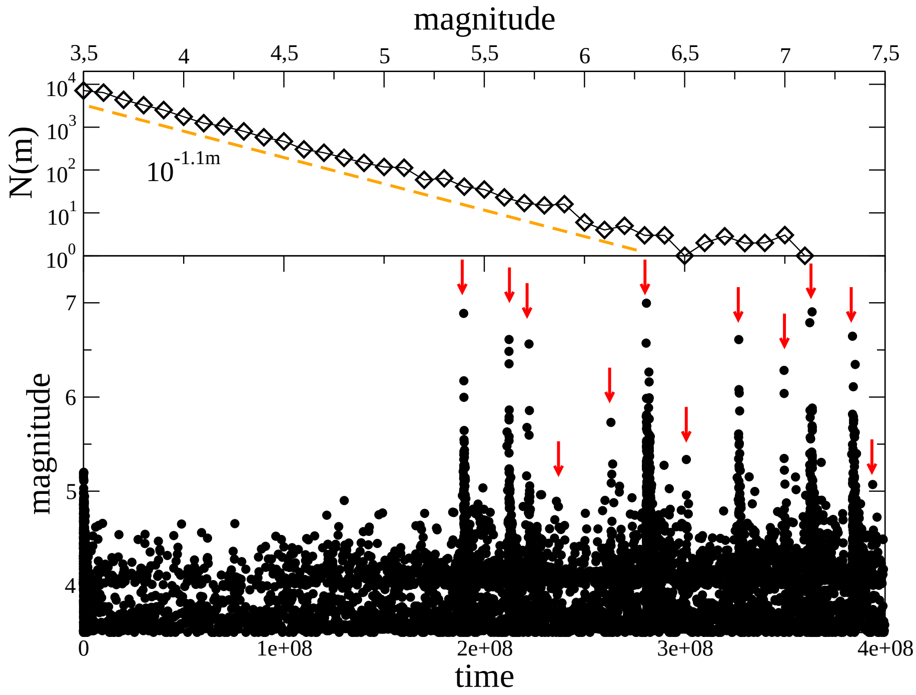

FIG. 4. (Upper panel) The magnitude-frequency distribution for the simulated catalog is compared with the GR law of experimental catalog with $b = 1.1$ (orange broken line). (Lower Panel) A typical temporal window within the seismic history generated by the model of Fig.3 of the manuscript starting from a random initial condition. Time is expressed in seconds. After a transient regime of about $2.E8$ sec the system exhibits main-aftershock sequences with spatio-temporal patterns very similar to experimental ones. Mainshocks are indicated by red vertical arrows.

V. THE ROLE OF THE DYNAMIC FRICTION COEFFICIENT

In supplem. Fig.5 we plot the number of aftershocks as function of the time from the mainshock for different values of the dynamic friction coefficient $\mu_D \in [0, 0.4]$. Results indicate that the c-value, as well as the p-value, are not affected by μ_D. The only effect of a larger μ_D is a larger level of the background rate which corresponds to a flat behavior of the aftershock number at large time distances. The above result appears in agreement with experiments performed at high velocity suggesting that, for very low dynamic friction, significant weakening only occurs when sufficient displacement is accumulated.

[1] P. Shearer, E. Hauksson, G. Lin, *Bull. Seismol. Soc. Am.* **95**, 904 (2005).
[2] F. Waldhauser, D. P. Schaff, *J. Geophys. Res.: Solid Earth* **113**, B08311 (2008).
[3] N.I.E.D., *National Research Institute for Earth Science and Disaster Prevention* (2012).
[4] A.E.I.C., *Alaska Earthquake Information Center, Catalog is available at* $http://www.aeic.alaska.edu/html_docs/db2catalog.html$ (2011).
[5] I.S.I.D.E., *Italian Seismological Instrumental and Parametric Data-Base, Catalog is available at* $http://iside.rm.ingv.it/iside/standard/$ (2012).
[6] T. Utsu, A. Seki, *J. Seismol. Soc. Jpn.* **7**, 223 (1954).
[7] Y. Ogata, *Ann. Inst. Math.Statist.* **50**, 379 (1998).
[8] A. Helmstetter, D. Sornette, *Phys. Rev. E* **66**, 061104 (2002).
[9] M. Bottiglieri, L. de Arcangelis, C. Godano, E. Lippiello, *Phys. Rev. Lett.* **104**, 158501 (2010).
[10] Y. Y. Kagan, *Bulletin of the Seismological Society of America* **94**, 1207 (2004).
[11] A. Helmstetter, Y. Kagan, D. Jackson, *Bull. Seism. Soc. Am.* **96(1)**, 90 (2006).

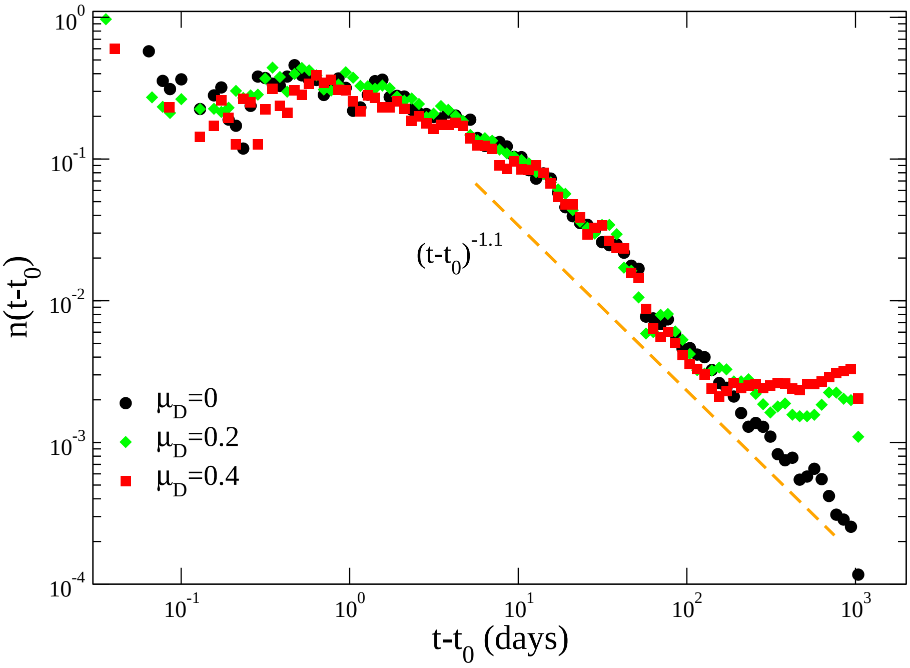

FIG. 5. The number of aftershocks as function of the temporal distance from the mainshock for different values of the friction coefficient μ_D.
